# Chemical Analysis, Distributed Modelling and Risk Indices. Three Fundamental Pillars in Risk Assessment

**DOI:** 10.1100/tsw.2002.858

**Published:** 2002-06-13

**Authors:** Emilio Benfenati, Daniel Genah, Roberto Verro, Paulo Mazzatorta

**Affiliations:** Department of Environmental Health Science, Istituto di Ricerche Farmacologiche Mario Negri, Via Eritrea 63, Milano, Italy; Montanagis S.r.l., Corso XXII Marzo 19, Milano, Italy

**Keywords:** risk assessment, chemical analysis, distributed models, risk indices, GIS

## Abstract

The Risk Assessment (RA) of pollutants from contaminated sites and landfills is a major environmental issue in Europe, due to the large number of sites and to the importance of groundwater protection. The high number of contaminants and the lack of knowledge of their environmental properties and their ecotoxicological and toxicological characteristics complicate the problem. Furthermore, this information about the chemicals has to be combined with the data relative to the territory and to the targets. We will describe the problems relative to each topic involved in the process, and we will propose an integrated methodology for coping with these problems, using state-of-the-art approaches in each part of the protocol. This methodology has been applied in several real cases.

## INTRODUCTION

In the last 10 years ecotoxicological and toxicological Risk Assessment (RA) methodologies became more and more important in the E.U. and the U.S. as fundamental instruments in environmental management[1,2,3]. The recent Directive on Landfills[2] gives a general frame in which activities should be focused, but the measures to protect the environment need more detailed operative tools.

The problem is multidisciplinary and cannot be solved without a robust analysis of all involved topics. The initial point should be a careful examination of the pollutant potential of landfills or contaminated sites. Necessary information on the territory has to be found and collected, defining all the sensitive targets. Other necessary information refers to ecotoxicological, toxicological, and environmental properties of the pollutants.

Keeping in mind all these topics, we developed a procedure that allows risk prediction for human beings and for the environment, due to contaminated sites and, more specifically, to landfills. Moreover, in the case of landfills, RA can be predictive, in the hypothesis of a potential leak.

## MATERIAL AND METHODS

The general flowchart of the developed approach is shown in Fig. 1[4]. The approach consists of a procedure that includes multidisciplinary activities: chemical analysis, territorial and environmental studies, flux and transport modelling, Predicted Environmental Concentration (PEC) and Total Daily Intake (TDI) calculation, and quantitative risk indices application.

The principal pollution transport vehicle from the source to the targets has been identified and limited, in the landfill case, to groundwater. This choice depends on the fact that, in many cases, this compartment is the most affected.

The *preliminary phase*, in the methodology, represents the base of the procedure. In this phase chemical and environmental data must be selected and collected; ways in which risk can arise have to be individuated and organised in a Conceptual Model[4].

The chemical information represents the first necessary step to evaluate the risk posed by pollutants[6]. Working on landfills, we focused our attention on the leachate. This is the material that can contaminate the groundwater, and it is simpler than the raw solid waste.

Detailed chemical analyses have to be done, allowing characterisation of the pollutants. Special attention has to be paid to organic pollutants, which are usually not sufficiently analysed[5,6,7]. To do this, GC–MS is a necessary technique, but LC–MS is recommended for integration of polar pollutants. Hundreds of pollutants can be found in a leachate with this approach.

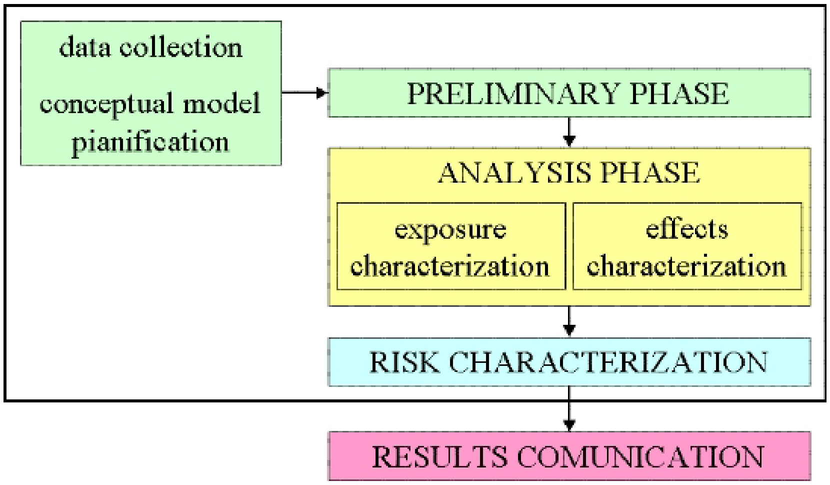

**FIGURE 1.** Methodology flowchart.

On the basis of the list of the pollutants found, environmental, ecotoxicological, and toxicological information must be collected to characterise the pollutants relative to the territory and targets. Data for a substance characterisation[8] are normally divided in three classes:

1. Physico-chemical properties and interaction with groundwater, such as Log $K_{ow}$, Log Koc, Kd, and R.
2. Ecotoxicological data, such as toxicity to algae, *Daphnia magna*, fish, plants, worms, birds, and mammals.
3. Toxicological data as NOAEL (No Observed Adverse Effect Level), LOAEL (Lowest Observed Adverse Effect Level), ADI (Acceptable Daily Intake), and RfD (Reference Dose).

These data can be found in appropriate databases or literature sources.

Some software packages can be used to obtain ecotoxicological missing values[9,10,11]. In this case, and for toxicity prediction, specific criteria have to be fulfilled[10].

Legislative limits can be used for the main inorganic pollutants[2,12], while databases such as RTECS (Registry of Toxic Effects of Chemical Substances), IUCLID (International Uniform Chemical Information Database), and ECDIN (Environmental Chemicals Data and Information Network) or data in the literature can be used for other cases. In a similar way, data for ecotoxicological properties can be found in databases[13] or literature, or can be predicted. Targets are both aquatic and terrestrial organisms.

To reach the goal of the RA, all territorial and environmental data have to be collected. The information that necessarily has to be collected is:

- The characteristics of the site, such as aquifer type, aquifer depth, spatial relation with river bodies, and meteorological characteristics;
- The presence of wells next to the site and the relevant uses of the water;
- Relevant territorial elements such as distribution and population density in the area next to the site;
- Possible exposure pathways for the potential targets.

Territorial and environmental data necessary to achieve an RA are the output of several processes such as:

- Detailed geological and hydrogeological characterisation of the area influenced by the site;
- Analysis of the presence of wells and groundwater pumping rate and relative cartography;
- Superficial water bodies description;
- Natural environment description;
- Historical cartography analysis;
- Resident population and antropic activities distribution analysis;
- Railways, traffic networks, and principal transport ways description;
- Groundwater, superficial water, ecosystems, and area analysis.

The best way to manage all the collected information is to use a geographical information system (GIS)[14], based on raster and vector data format. The GIS is used to collect, store, retrieve, and elaborate input data for flux- and transport-distributed groundwater models (permeability, aquifer bottom elevation, porosity, organic carbon, recharge rate, etc.) and to manage model outputs. Since distributed groundwater model outputs[15,16] are represented by a set of a single geographical information layer for each time step in which the simulation is sliced, the GIS allows overlaying the spatial distribution of groundwater pollution with the topographic map. This allows identification of the intermediate targets as well, river/ditches, or exposed, quantification of pollutant concentration in water, and identification of how long a pollutant takes to reach the single intermediate target.

Collected data have to be used in the *Conceptual Model* construction[4]. A Conceptual Model consists of a written description and flowcharts of the foreseen relationships between the pollution source and identified targets.

Fundamental operations that have to be done are:

1. Risk Hypothesis project. A Risk Hypothesis consists of the sum of the relations between the pollution source and a target. The relations are related to the source and target characteristics.
2. Diagram realisation. Diagrams have to show in a graphic way the relations introduced in the Risk Hypothesis.

Since the groundwater is identified as the unique vector of transport, distributed models (both flux and transport) are needed to get the spatial and temporal distribution assessment of pollution in a model domain, in the exposure characterisation phase.

A hydrodynamic simulation mathematic model is an instrument that describes, in a quantitative way, the physical behavior of groundwater, depending on the local hydrogeological situation (hydrogeological cycle, underground hydrodynamics) and on the elements the mass balance consists of.

There are numerous examples in literature of models, which can be classified in four large groups:

- ***Sand box*****:** a model, created in the 1950s, considering a box full of soil and with a water flux;
- ***Analogic*****:** mostly used in the 1950s and 1960s, principally electric; they are based on the laws that control electricity and hydrogeology; some of these model are specific for liquid-viscous fluids;
- ***Deterministic or numeric models*****:** consider physical laws that control the hydrological cycle;
- ***Probabilistic models*****:** do not consider the physical point of view of the phenomenon, but try to determinate its behavior from a probabilistic point of view.

Nowadays, numeric or deterministic models are the most developed, because they allow simulating complex phenomena typical of hydrogeology. Deterministic or numeric models can be divided in two types: “black box” and “continuity equation based”. The “continuity equation based” are based on the conservation principle and on a mathematical relation that links the piezometric level to the parameters controlling groundwater flow. In the numerical models, the mathematical relations that describe the phenomena are expressed by equations solved with numerical calculation methods; the most famous numerical calculation methods are the finite-difference and finite-element methods. In each case, the studied area is divided by means of a geometric system characterised by a grid and relative cells or "nodes". The finite-difference method replaces the continuous ones with a set of discrete points arranged in a grid pattern; that means that the area is divided into a set of regular cells (square or rectangular), and the parameter’s value, as transmissivity or storability, is attributed to every cell — in particular to its centre if the calculation code is the "Block Centre Flow" one.

The finite-difference equation is solved using an iterative method. The Laplace equation is solved for each node on the basis of values at the surrounding four/six nodes.

With the finite-difference method, the boundary conditions govern the solutions; in fact, the results of the equations are processed starting from the same conditions.

For the solution of the transport problems, either the Eulerian methods or Lagrangian ones are used. The Eulerian approach is generally indicated in situations in which dispersion and chemistry reactions are prevalent; in situations in which advection is prevalent, the Eulerian method is subjected to numerical dispersion.

The Lagrange approach supplies a more accurate solution for the advection problems with high concentration gradient, even if working with an aquifer with large difference in the properties' values, where calculation problems can be generated by many sources.

To overcome this problem, a Eulerian-Lagrangian hybrid method code for transport simulation is produced; in this method, the relative terms of dispersion and chemical reactions are solved with the finite-difference method (Block Centred Flow), and the advection term is solved with the Lagrangian Method of the Characteristics (MOC).

In the MOC, a set of representative particles is generated in the finite-difference grid at the beginning of simulation, either randomly or following a fixed pattern. A concentration is assigned to each particle that is equal to the concentration of the cell in which it is initialised. Each particle is associated with a set of attributes, including the spatial coordinates and the concentration. During the simulation, the particle moves forward through the flow, and at every step, the concentration in each cell is calculated; average concentrations in the cells are calculated as well. This way, it is possible to get a concentration in time and space for every chemical element or compound.

The coupling between distributed models and GIS is an effective way to get information about the identification and quantification of the pollution and the timing of its transport to the intermediate target.

The transport modelling results are used, in the exposure-characterisation phase, to calculate PECs (for ecosystems) and TDIs (for human beings). PECs and TDIs are calculated using specific equations[17] that allow quantity estimation of each substance reaching a specific target through every compartment, as shown in the Conceptual Model.

A crucial point in the developed methodology consists of using specific indices for toxicological and ecotoxicological *risk characterisation*[18]. RA, as normally done, ends with the comparison of studied substance concentration in groundwater and legislative limits for the same substance. A more modern method consists in the comparison between PECs and toxicity value for a substance. Thus, a Toxicological Exposure Ratio (TER) is obtained. But it is also possible to integrate different TERs (one for every organism representative for a certain ecosystem) into an index.

The definition of suitable *quantitative risk indices*, both for human beings and for ecosystems is obtained by advanced ecotoxicological studies[18]. The work includes the previous identification of the environmental compartment mostly at risk and the identification of organisms representative of the environmental systems. Then it is necessary to calculate a TER for each bioindicator; a partial score for each TER, dependent on the role the bioindicator has in the particular ecosystem it belongs to, is given. By the combination of all weighted partial scores, through a mathematical algorithm for final synthetic score and an interactive process on the list of chemicals previously selected, it is possible to finally calibrate the risk indexes.

In the case of human beings, integrated indexes are used too[17]. In this case the index is obtained by the product between a factor representing the hazard and a factor representing the persistence of the substance. The hazard results from the rate between the TDI (total daily intake) representative of the exposure and the ADI (admissible daily intake) representative of the effects.

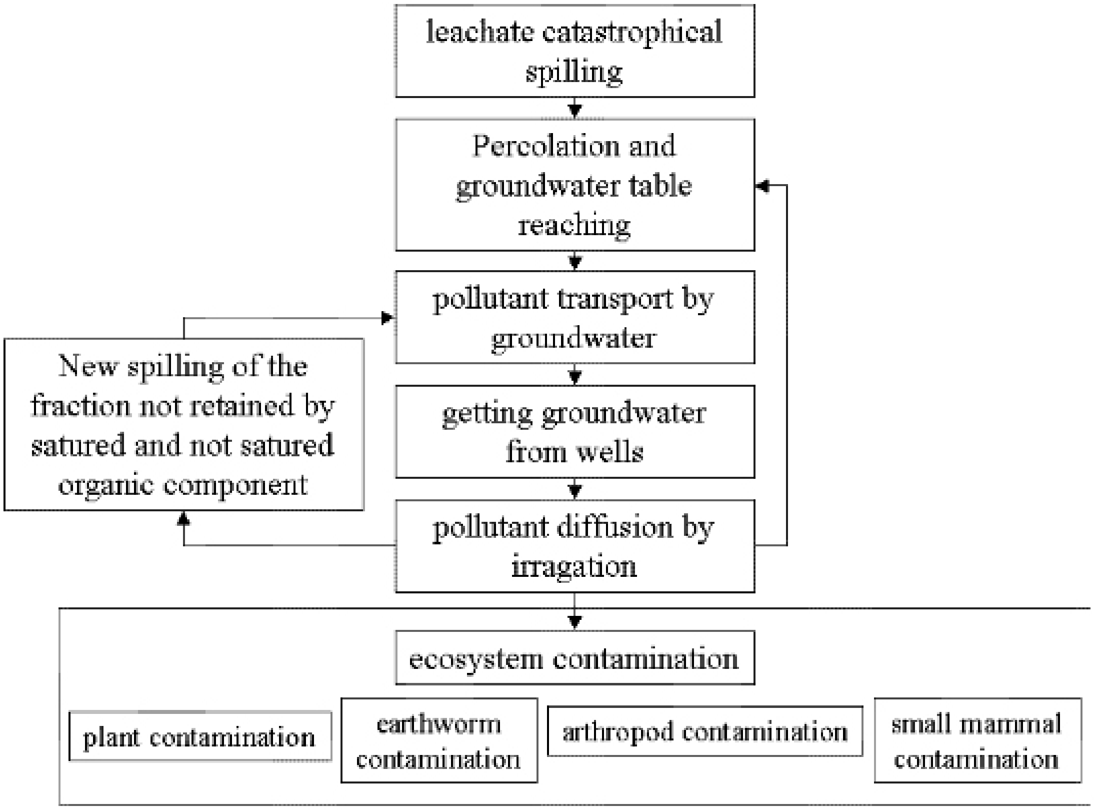

**FIGURE 2.** Simplified Risk Hypothesis diagram for the terrestrial ecosystem.

## THE REAL CASE

We applied our methodology to several cases. The particular case shown here refers to a controlled landfill. We assessed the risk resulting from a leachate leak in a worst case that consisted of several days leachate production reaching groundwater as if no protection, neither artificial nor natural, existed.

Data collection was done, and we identified specific targets on the territory that could be involved in this hypothetical contamination. Then, we developed a Risk Hypothesis for them. Identified targets were resident population, workers, and terrestrial and aquatic ecosystems. Fig. 2 represents the example of a simplified Risk Hypothesis diagram for the terrestrial ecosystem.

We studied the aquifer characteristics, and we modelled the transport for several substances. Fig. 3 shows three steps of the arsenic (one of the modelled substances) transport. The transport modelling indicated that no risk could be foreseen for the aquatic ecosystem and for resident population. On the contrary, risk was possible for farmers and for the terrestrial ecosystem of cultivated fields.

After applying equations for TDI and PEC calculation, we used the obtained values in risk indexes, both for human being and for the terrestrial ecosystem. Fig. 4 shows, in a very simplified way, the results obtained for the population working on fields. Different coloured lines represent, for all the modelled substances, different risk levels reached within 50 years.

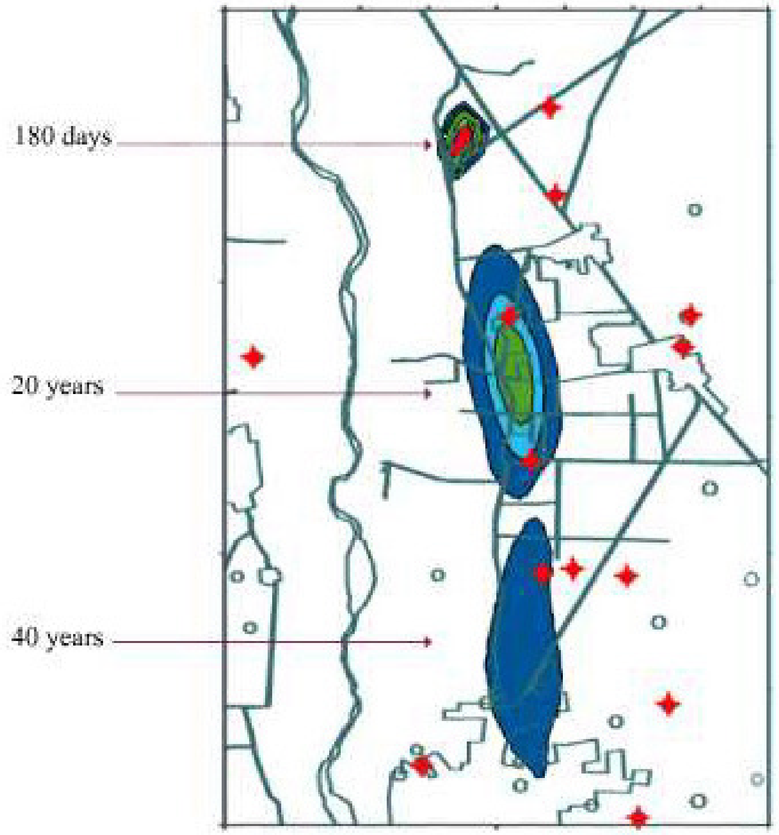

**FIGURE 3.** Three steps of the arsenic transport modelling.

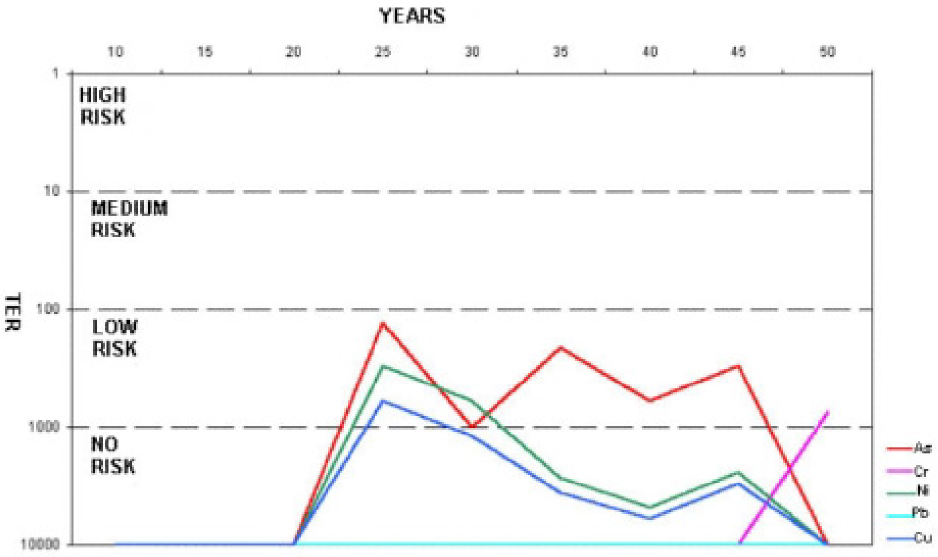

**FIGURE 4.** Risk index results obtained for the population working on fields.

## CONCLUSIONS

The proposed methodology is effective for assessing site-specific risks of contaminated sites, in particular landfills, taking into account the entire environmental system.

The principal innovations of the methodology are listed below:

- First, the methodology allows focussing the attention on final and specific targets. Targets are final targets because they are human beings or ecosystems, instead of intermediate targets such as wells or irrigation channels; they are site-specific targets because they really exist in the real examined scenario.
- Second, the methodology is GIS based. This means that it is possible to manage as much territorial data as necessary as input data, and it is possible to reference to the territory all the methodology outputs.
- Third, in the ecosystem-specific case, we tried to reproduce the organism multiplicity and their mutual relations, with the application of integrated risk indices. To cope with this objective the coupling between GIS and distributed models was necessary.

## ACKNOWLEDGEMENTS

This paper was presented at the CSIS/ESF Workshop, Analysis, Toxicity, and Biodegradation of Organic Pollutants in Groundwater from Contaminated Land, Landfills, and Sediments, Barcelona, Spain, 8–10 November 2001.
